# Point-of-care CD4 technology invalid result rates in public health care settings across five countries

**DOI:** 10.1371/journal.pone.0219021

**Published:** 2019-07-05

**Authors:** Katherine Lamp, Seth McGovern, Youyi Fong, Biruhtesfa Abere, Adisu Kebede, Gonfa Ayana, Achamyeleh Mulugeta, Chares Diko Atem, Jean Bosco Elat Nfetam, Divine Nzuobontane, Timothy Bollinger, Ilesh Jani, Nadia Sitoe, Charles Kiyaga, George Senyama, Phibeon Munyaradzi Mangwendeza, Sekesai Mtapuri-Zinyowera, Jilian A. Sacks, Naoko Doi, Trevor F. Peter, Lara Vojnov

**Affiliations:** 1 Clinton Health Access Initiative, Boston, Massachusetts, United States of America; 2 Fred Hutchinson Cancer Research Center, Seattle, Washington, United States of America; 3 Clinton Health Access Initiative, Addis Ababa, Ethiopia; 4 Ethiopian Public Health Institute, Addis Ababa, Ethiopia; 5 Clinton Health Access Initiative, Yaoundé, Cameroon; 6 National AIDS Control Committee, Yaoundé, Cameroon; 7 Clinton Health Access Initiative, Maputo, Mozambique; 8 Instituto N*acio*nal de Saúde, Maputo, Mozambique; 9 Central Public Health Laboratory, Kampala, Uganda; 10 Clinton Health Access Initiative, Kampala, Uganda; 11 Clinton Health Access Initiative, Harare, Zimbabwe; 12 Ministry of Health and Child Care, Harare, Zimbabwe; University of Ghana College of Health Sciences, GHANA

## Abstract

**Background:**

Since 2010, point-of-care (POC) CD4 testing platforms have been introduced in both urban and rural settings to expand access to testing by bringing diagnostic services closer to patients. We conducted an analysis of routinely collected CD4 testing data to determine the invalid result rates associated with POC CD4 testing.

**Methods:**

We analyzed 981,152 CD4 testing records collected from Alere Pima Analyzers between January 2011 and December 2016 across five countries in sub-Saharan Africa. Routinely collected data and programmatic records were used to determine the rate of invalid test results per month, by facility type, and by operator based on cumulative usage during the study period. In addition, frequency of invalid test types and utilization of control beads were assessed.

**Results:**

Across the five countries, 75,530 invalid messages were returned, resulting in an overall invalid result rate of 7.7%. The invalid result rate by country ranged from 6.6% to 11.2%. Invalid result rates were consistent across facility types. Invalid result rates were inversely correlated with operator usage: low volume operators (<50 tests over study period) experienced an invalid result rate of 10.2%, while high volume operators (>500 tests over study period) experienced an invalid result rate of 5.5%. Two invalid result types (exposure position control and reagent control) accounted for nearly 50% of invalid results. Routine data showed that control beads were run on 88.3% of days that the device was used.

**Conclusions:**

Our analysis found that the rate of invalid results was consistent across all types of health facilities, indicating that decentralization of POC CD4 testing to lower level health facilities did not exhibit high invalid result rates or increase cartridge wastage. Additionally, invalid result rates were inversely correlated to operator usage, with high-volume operators experiencing lower invalid result rates than low-volume operators. POC CD4 testing can, therefore, be performed in decentralized national testing programs; however, adequate training, quality assurance, routine monitoring, and ongoing mentorship should also be implemented for success.

## Introduction

CD4 enumeration has previously been used in resource-limited settings to determine treatment eligibility of HIV-positive patients and for routine monitoring to identify treatment failure and opportunistic infections [1,2]. Recommendations released by the World Health Organization (WHO) in 2016 eliminated the necessity for CD4 testing as a requirement to determine treatment eligibility and endorsed viral load testing as the preferred method to monitor treatment [3]. However, the WHO has recently re-affirmed the continued importance of CD4 testing for clinical patient and opportunistic infection management, identification of patients with advanced HIV disease, and in areas with limited access to viral load testing [4,5].

Considerable investments made in CD4 testing networks have resulted in sufficient testing capacity to provide CD4 access to all people living with HIV [6]. A 2013 review of CD4 testing capacity across 68 countries indicated that there is capacity to provide all people living with HIV 4.6 CD4 tests per year, yet only 13.7% of CD4 testing capacity was being utilized [6]. Despite the available testing capacity, access to CD4 testing is still poor for many people living with HIV. As of 2013 –when CD4 count was still being used to determine treatment eligibility–only 20% of people diagnosed with HIV received a CD4 test in some regions [7]. Conventional CD4 testing programs often require transporting samples to central laboratories in urban centers, resulting in long test turnaround times, high loss to follow up, and delayed treatment initiation.

Since 2010, point-of-care (POC) CD4 testing platforms have been introduced in over 50 countries to increase access to CD4 testing services [6,8]. These devices have lower daily testing throughput than conventional platforms (~20–80 tests per day vs. ~200) [9], but can be operated by facility-based, non-laboratory professional health workers and provide same-day test results to patients, allowing for faster clinical decision-making. A systematic review of POC CD4 testing found that these technologies reduced test turnaround time and increased retention along the HIV treatment cascade from HIV testing through ART initiation [10]. Furthermore, the use of POC CD4 testing was found to increase life expectancy, and to be cost-effective and acceptable to both patients and providers [10].

A key challenge associated with decentralized POC CD4 testing is the need to monitor a large number of devices and operators across an extensive testing network. To overcome this, national programs can utilize the benefit of connectivity with POC device-based technologies, defined as the ability of a device to successfully transmit data to a centralized system, ideally at least once per day [11]. Variables transmitted via connectivity may include daily testing volumes, internal quality control (IQC) results, invalid codes, etc. As part of a comprehensive quality assurance system, connectivity is a key component in providing Ministries of Health the information they need for time-sensitive decision-making and management of diagnostic networks. We used testing data collected by POC CD4 devices in five countries in sub-Saharan Africa to determine the proportion of invalid results during routine use and to demonstrate the analyses that can be conducted using routinely collected data to support quality improvement efforts for POC testing.

## Methods

We conducted a retrospective, observational, cross-sectional analysis of routine testing data from five countries in sub-Saharan Africa (Cameroon, Ethiopia, Mozambique, Uganda, and Zimbabwe) using the Alere Pima Analyzer. Testing data captured between January 2011 and December 2016 were included for analysis, however start dates varied by country based on when the Alere Pima Analyzer was introduced nationally.

The Alere Pima Analyzer automatically records data about the device, test date and time, and numerical CD4 test outcome. Additional information can be added in free text fields for operator and/or patient identification. The Alere Pima Analyzer has the capability to transmit data collected by the device wirelessly via the cellular SMS/GPRS network to a centralized database. The majority of the data used in this analysis was transmitted via cellular network; however some records may have been extracted manually from the analyzers using a flash drive and uploaded directly to the database for facilities with poor network coverage. Once transferred, the testing data can be stored either on the device manufacturer’s proprietary data hosting and visualization service, Data Point, or on a local database. In Cameroon, Mozambique and Uganda the Pima testing data was transmitted to Data Point, and in Ethiopia and Zimbabwe it was transmitted to a government-owned, local server and dashboard. From either type of database, the same raw testing data can be downloaded into a CSV file for analysis.

The Alere Pima Analyzer’s internal quality control (IQC) function aborts the testing process if an invalid result is detected due to the device, reagent cartridge, or sample–returning an invalid message instead of a numeric CD4 result. Testing records were provided from each country’s central POC CD4 database with each record including the following variables: unique device ID number, assay (CD4 test or control bead), CD4 count (if successful), invalid message (if encountered), coded invalid number (if encountered), operator name (if available), result date, test start and end times, IQC checks, test ID number, and software version.

The rate of invalid test results was disaggregated by country, facility type, operator, and across time. The rate of invalid test results was determined by dividing the total number of invalid tests by the total number of tests run (control beads excluded). For the facility type analysis, the device identification number was matched with programmatic records to assign the facility name, district, and region where the device was located if this was not already included in the national database. The types of facilities were categorized as hospitals, health centers, clinics, other (prisons or laboratories), and unknown (devices that did not have a facility listed in database or programmatic records, or the facility type could not be discerned from the given available name). Facility type invalid result rates were calculated using the median of individual facility invalid result rates. Unique operators were identified by combining the operator name, if provided, with the facility name. The total number of tests conducted by each Operator-Facility combination was determined, and the operator was assigned to a testing threshold. Additionally, analysis of the frequency of each invalid type was also conducted. Each invalid message can be attributed to one or more likely cause(s): device, operator, reagent or a combination of these. The likely cause was determined using the Alere Pima Error Resolution Guide, which includes a description of each invalid result message to guide service and repair activities [12]. Finally, data related to utilization of control beads were analyzed as well: the proportion of testing days in which only control beads were run, only CD4 tests were run, or both control beads and CD4 tests were run.

Data analysis was conducted using Microsoft Excel 2010 v.14.0.7183.5000 (Redmond Washington) and the R statistical programming language and environment.

This study was approved by each country’s Institutional Review Board: study was approved by each country’s Institutional Review Boards: Cameroon National Ethical Committee of Research for Humans, Ethiopian Public Health Institute Institutional Review Board, Mozambique’s National Health Bioethics Committee, Uganda’s Makerere University Institute of Public Health Higher Degrees, Research and Ethics Committee, Medical Research Council of Zimbabwe as well as the US-based Advarra IRB.

## Results

A total of 981,152 CD4 test records were captured and included for analysis. Of these, 905,622 CD4 tests passed the device’s IQC checks and returned valid CD4 test results, while 75,530 were aborted and returned an invalid message (Table 1). A total of 938 facilities (median per country = 123) and 1,094 devices (median per country = 148) conducted CD4 testing. In total, 8,204 unique operators were recorded as having run tests (median per country = 2,080) in Cameroon, Mozambique, Uganda and Zimbabwe. Due to national data sharing policies in Ethiopia, operator names were not provided and therefore not included in the operator analyses. The overall invalid result rate during the study period was 7.7% (95% CI: 7.65–7.75%). Invalid result rates during the study period per country were: 6.6% in Mozambique, 7.2% in Ethiopia, 7.7% in Cameroon, 8.7% in Zimbabwe, and 11.2% in Uganda.

**Table 1:** Summary of Pima CD4 testing volumes and invalid results by country.

Country	Number of Health Facilities with Pima Devices		Number of Alere Pima Analyzers		Number of Device Operators		Operators per Facility	Tests per Operator	Number of CD4 Tests Run (2011–2016)		Number of Invalid Results		Invalid Result rate
	n	%	n	%	n	%	Median (IQR)	Median (IQR)	n	%	n	%	% (95% CI)
Cameroon	123	13.1%	148	13.5%	774	9.4%	6 (4)	6 (34)	41,730	4.3%	3,224	4.3%	7.7% (7.5–8.0%)
Ethiopia	78	8.3%	78	7.1%	-	-	-	-	44,424	4.5%	3,191	4.2%	7.2% (7.0–7.4%)
Mozambique	208	22.2%	276	25.2%	3,252	39.6%	12 (14)	9 (115)	643,567	65.6%	42,374	56.1%	6.6% (6.5–6.6%)
Uganda	432	46.1%	488	44.6%	3,270	39.9%	5 (6)	13 (55)	197,175	20.1%	22,013	29.1%	11.2% (11.0–11.3%)
Zimbabwe	97	10.3%	104	9.5%	908	11.1%	8 (6)	13 (55)	54,256	5.5%	4,728	6.3%	8.7% (8.5–9.0%)
**Overall**	**938**	**100%**	**1,094**	**100%**	**8,204**	**100%**	**7 (7)**	**11 (64)**	**981,152**	**100%**	**75,530**	**100%**	**7.7% (7.7–7.8%)**

The lowest invalid result rate, 6.0% (95% CI: 5.8–6.1%), occurred in 2013 (Table 2). In 2011, fewer than 150 tests were run across the five countries; therefore, excluding 2011, the highest invalid result rate, 11.3% (95% CI: 10.9–11.8%) occurred in the first year of full implementation, 2012. The invalid result rate in 2016 was 8.0% (95% CI: 7.9–8.1%).

**Table 2:** Pima CD4 testing volumes and invalid results per year.

Year	Number of CD4 Tests Run (2011–2016)		Number of Invalid Results		Invalid Result rate
	n	%	n	%	% (95% CI)
2011	149	0.0%	39	0.1%	26.2% (19.8–33.8%)
2012	18,143	1.8%	2,056	2.7%	11.3% (10.9–11.8%)
2013	99,892	10.2%	5,987	7.9%	6.0% (5.8–6.1%)
2014	173,037	17.6%	10,481	13.9%	6.1% (5.9–6.2%)
2015	330,939	33.7%	28,198	37.3%	8.5% (8.4–8.6)%)
2016	358,992	36.6%	28,769	38.1%	8.0% (7.9–8.1%)
**Overall**	**981,152**	**100%**	**75,530**	**100%**	**7.7% (7.6–7.8%)**

Facilities were grouped into five categories to determine the percentage of tests run at each level: hospital (15.5%), medical center (2.6%), health center (71.9%), clinics (1.4%), other (0.2%), and unknown facilities or facility level (8.5%) (Table 3). Despite health centers and hospitals running the majority of tests, the median rate of invalid results was consistent across facility levels within each country. Health centers and hospitals had median invalid result rates of 9.4% (95% CI: 6.1–14.0%) and 8.6% (95% CI: 6.1–13.4%), respectively, while clinics had a median invalid result rate of 7.9% (95% CI: 6.0–13.1%).

**Table 3:** Total tests run and invalid results and rates by health care facility type per country.

Facility Level	Total Facilities		Number of CD4 Tests Run (2011–2016)		Number of Invalid Results		Median Error Rate	p-value
	n	%	n	%	n	%	% (IQR)	
**Cameroon**								
*Hospital*	65	53%	23,328	56%	1,756	54%	7.4% (4.7–13.2%)	Ref.
*Medical Center*	12	10%	3,823	9%	272	8%	5.9% (3.2–12.3%)	0.388
*Health Center*	15	12%	4,161	10%	283	9%	7.5% (4.0–15.0%)	1.000
*Clinic*	1	1%	140	0%	6	0%	4.3% (4.3–4.3%)	0.319
*Other*	-	-	-	-	-	-	-	-
*Unknown*	30	24%	10,278	25%	907	28%	10.9% (6.6–17.6%)	0.117
**Ethiopia**								
*Hospital*	3	4%	553	1%	105	3%	17.7% (14.7–18.7%)	Ref.
*Medical Center*	-	-	-	-	-	-	-	-
*Health Center*	27	35%	15,125	34%	1,005	31%	9.0% (5.1–13.5%)	0.860
*Clinic*	-	-	-	-	-	-	-	-
*Other*	1	1%	51	0%	8	0%	15.7% (15.7–15.7%)	1.000
*Unknown*	47	60%	28,695	65%	2,073	65%	7.1% (4.2–11.4%)	0.041
**Mozambique**								
*Hospital*	17	8%	75,702	12%	5,581	13%	7.1% (4.8–8.1%)	Ref.
*Medical Center*	-	-	-	-	-	-	-	-
*Health Center*	151	73%	513,349	80%	31,997	76%	6.4% (4.6–8.2%)	0.748
*Clinic*	14	7%	21,086	3%	1,800	4%	9.0% (7.5–12.0%)	0.084
*Other*	-	-	-	-	-	-	-	-
*Unknown*	26	13%	33,430	5%	2,996	7%	10.1% (7.4–16.0%)	0.0009
**Uganda**								
*Hospital*	54	13%	37,880	19%	4,416	20%	11.8% (8.1–14.5%)	Ref.
*Medical Center*	-	-	-	-	-	-	-	-
*Health Center*	363	84%	146,608	74%	16,377	74%	11.4% (8.0–16.4%)	0.871
*Clinic*	5	1%	1,150	1%	160	1%	13.5% (13.1–14.0%)	0.391
*Other*	3	1%	622	0%	59	0%	12.5% (7.2–14.8%)	0.929
*Unknown*	7	2%	10,915	6%	1,001	5%	5.9% (4.7–8.7%)	0.028
**Zimbabwe**								
*Hospital*	27	28%	14,139	26%	1,195	25%	7.1% (6.0–10.4%)	Ref.
*Medical Center*	-	-	-	-	-	-	-	-
*Health Center*	43	44%	27,052	50%	2,384	50%	7.4% (6.2–12.7%)	0.522
*Clinic*	26	27%	12,118	22%	1,106	23%	7.8% (5.9–11.0%)	0.742
*Other*	1	1%	947	2%	43	1%	4.5% (4.5–4.5%)	0.286
*Unknown*	-	-	-	-	-	-	-	-
**Overall**								
*Hospital*	166	18%	151,602	15.5%	13,053	17%	8.6% (6.1–13.4%)	Ref.
*Medical Center*	12	1%	3,823	2.6%	272	0%	9.3% (5.2–13.0%)	0.237
*Health Center*	599	64%	706,295	71.9%	52,046	69%	9.4% (6.1–14.0%)	0.413
*Clinic*	46	5%	34,494	1.4%	3,072	4%	7.9% (6.0–13.1%)	0.863
*Other*	5	1%	1,620	0.2%	110	0%	12.5% (4.5–15.7%)	0.909
*Unknown*	110	12%	83,318	8%	6,977	9%	8.6% (5.3–14.9%)	0.964
**Overall**	**938**	**100%**	**981,152**	**100%**	**75,530**	**100%**		

Out of the 8,204 unique operators identified, 5,865 (71.5%) ran fewer than 50 tests over the study period, 728 (8.9%) ran 51–100 tests, 638 (7.8%) ran 101–200 tests, 554 (6.8%) ran 201–500 tests, and 419 (5.1%) ran over 500 tests (Table 4). Despite the majority of operators running fewer than 50 tests, these low volume operators conducted just 6.4% of all tests. Operators who ran more than 500 tests conducted 58.8% of the total testing volume. Invalid result rates were inversely related to operator usage, with the lowest volume operators (<50 tests) having an invalid result rate of 10.2% (95% CI: 10.0–10.5%) compared to high volume operators (>500 tests), who had an invalid result rate of 5.5% (95% CI: 5.4–5.5%).

**Table 4:** Total tests run and invalid results and rates by operator testing volume.

Number of Tests Performed Per Operator	Number of Operators		Number of Tests Conducted		Number of Invalid Results		Invalid Result Rate
	n	%	n	%	n	%	% (95% CI)
1–50	5,865	71.5%	58,307	6.4%	5,975	9.8%	10.2% (10.0–10.5%)
51–100	728	8.9%	52,375	5.7%	4,719	7.7%	9.0% (8.8–9.3%)
101–200	638	7.8%	91,858	10.1%	8,340	13.7%	9.1% (8.9–9.3%)
201–500	554	6.8%	173,644	19.0%	12,718	20.8%	7.3% (7.2–7.4%)
>500	419	5.1%	536,186	58.8%	29,319	48.0%	5.5% (5.4–5.5%)
**Overall**	**8,204**	**100%**	**912,370**	**100%**	**61,071**	**100%**	

**Fig 1:**
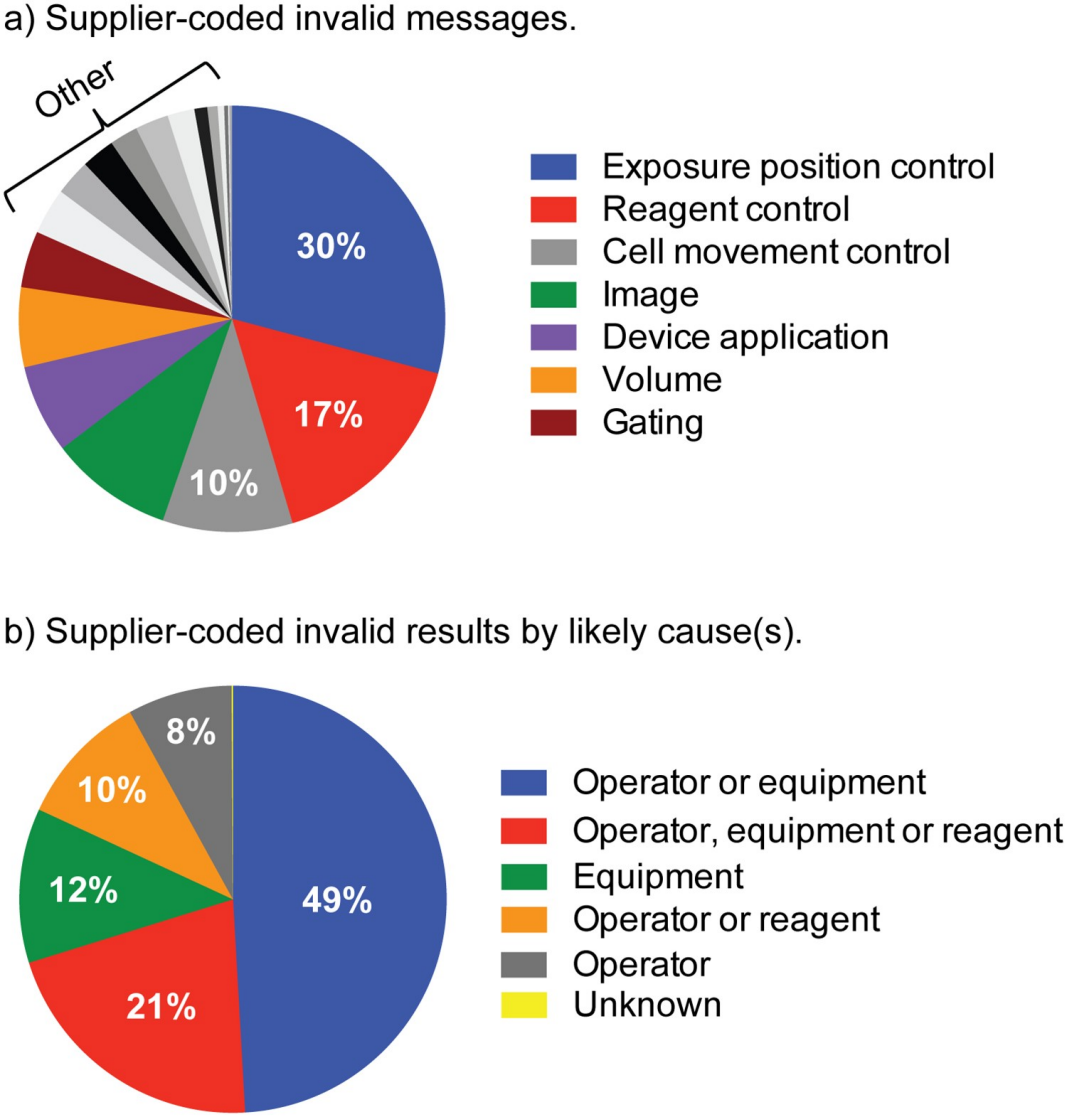
Supplier-coded invalid messages (a) and invalid results by likely cause(s) (b).

Each time a CD4 test is run, the IQC system within the Alere Pima Analyzer checks the cartridge barcode, reagent expiration date, sample volume, device optics and reagents. The device then returns either a numeric CD4 count or an invalid message and specific invalid code, which can be attributed to a likely cause: operator, equipment, reagent or a combination of the three. The most common invalid test messages observed were *Exposure position control—Invalid Test Error 850* (29.9%), *Reagent control—Invalid Test Error 860* (16.7%), C*ell movement control—Invalid Test Error 910* (10.1%), *Image—Invalid Test Error 880* (9.6%), *Device application*—*Invalid Test Error 210* (6.9%), and *Volume—No Sample Detected Error 201* (6.2%) (Fig 1a). All other invalid messages occurred in less than 4.5% of invalid results. 49.1% of invalid results could be attributed to the operator or equipment, 22.1% to the operator, equipment or reagent, 10.7% to the equipment, 10.1% to the operator or reagent, 7.9% to the operator, and 0.1% unknown (Fig 1b).

In addition to analyzing invalid result rates for CD4 tests, we looked at the pattern of control bead utilization. Overall, Alere Pima Analyzers were utilized on 171,263 days across the study period and countries. Control bead runs were recorded on 151,229 (88.3%) of the days within which the devices were used, while CD4 tests were run on 148,053 days (86.4%). There were 20,034 days (11.7%) when a CD4 test was conducted but no control bead was run (Table 5).

**Table 5:** Control bead and CD4 testing run days by country and all countries combined.

Country	Total Days of Pima Use	Days with Only Beads Run		Days with Only CD4 Tests Run		Days with Both Beads and CD4 Tests Run		Total Days of Control Bead Use		Total Days of CD4 Testing	
	n	n	%	n	%	n	%	n	%	n	%
Cameroon	15,220	1,337	8.8%	985	6.5%	12,898	84.7%	14,235	93.5%	13,883	91.2%
Ethiopia	14,804	3,182	21.5%	2,882	19.5%	8,740	59.0%	11,922	80.5%	11,622	78.5%
Mozambique	77,418	6,310	8.2%	3,002	3.9%	68,106	88.0%	74,416	96.1%	71,108	91.8%
Uganda	48,245	9,189	19.0%	10,739	22.3%	28,317	58.7%	37,506	77.7%	39,056	81.0%
Zimbabwe	15,576	3,192	20.5%	2,426	15.6%	9,958	63.9%	13,150	84.4%	12,384	79.5%
**Overall**	**171,263**	**23,210**	**13.6%**	**20,034**	**11.7%**	**128,019**	**74.7%**	**151,229**	**88.3%**	**148,053**	**86.4%**

## Discussion

The overall rate of invalid results across the five countries was 7.7%, while the invalid result rates per country ranged from 6.6% to 11.2%. This analysis was unable to determine the causes for the inter-country variation; however, they may be due to differences in the type of training provided to operators, level of monitoring or supervision offered at each site, funding availability for the POC CD4 program or level of corrective actions taken to reduce invalid result rates across the five countries. Invalid result rates were consistent across different levels of health facilities, from clinics to hospitals, indicating that decentralization of testing to lower level facilities did not does not result in an increase in invalid result rates or cause increased cartridge wastage due to invalid results. These results are indicative of the high quality of testing services that can be provided through a decentralized POC testing network.

The invalid result rates observed in this study are in line with the results seen in previous studies, which vary between 2% and 20% [13–17]. However, much of the previous literature was taken from diagnostic accuracy studies or from non-government run facilities. Small sample sizes or differences in operator training and quality assurance protocols may explain the variation in invalid result rates previously seen. The data in this analysis were taken from national public health programs across a number of countries and therefore may be more generalizable to other national government health programs.

The invalid result rate varied considerably based on operator usage, with high volume operators (>500 tests) returning a 5.5% invalid result rate and low volume operators (<50 tests) returning an invalid result rate of 10.2%. The majority of operators (71.5%) were responsible for running fewer than 50 tests over the study period analyzed, yet this group ran less than 10% of overall tests and experienced almost two times the invalid result rate of high volume operators. This indicates a large number of operators do not routinely use the devices and are not reaching proficiency. Comprehensive training programs that provide hands-on practice and include competency assessments may promote greater proficiency among all operators and reduce invalid result rates to those observed with the high volume operators. In addition, linking programmatic records on operator cadre, operator certification, training activities and/or supervision with the routine testing data captured by the POC CD4 device can help to identify operators in need of follow up mentorship and identify additional patterns in invalid rates. In order for this to be successful, data entry should be standardized to ensure that operator names are consistently entered to allow performance to be tracked over time and across sources.

Consistent use of control beads is essential for ensuring that devices produce accurate results across the entire range of CD4 counts. Analysis of control bead utilization showed that facilities were consistently running control beads. Our study found that control beads were run on 86.4% of days in which a CD4 test was run. This was similar to control bead utilization seen in previous studies: one study found that control beads were used on 96.62% of CD4 testing days [14], while another that found controls beads were used on 86.91% of CD4 testing days [17]. Although the use of control beads on their own is not sufficient to improve the quality of testing and ensure that devices are properly calibrated, consistent bead use does suggest that devices were being routinely monitored at the site level (and standard operating procedures for daily quality control were followed).

Availability of routine testing data transmitted via wireless connectivity alone will not reduce the invalid result rate or improve testing quality. However, these data can be used by program managers to identify trends in invalid rates and bead usage, and when linked with programmatic data, target corrective action. Use of routine data captured on a centralized database can be maximized through regular review of the database and testing dashboards by dedicated program managers, generation of routine reports shared at the national and/or regional levels, and alerts that automatically flag changes in invalid rates or bead usage. In combination with programmatic data, as well as robust training programs, external quality assessments, and ongoing mentorship, connectivity data can allow POC program managers to reduce invalid rates and cartridge wastage to establish efficient, quality decentralized POC testing programs.

This study has several limitations. First, due to the lack of unique patient identifiers it was not possible to decipher distinct patients and some repeat testing may be included, although this was likely minimal and the results unaffected. Further, it was not possible to discriminate patients being tested to determine treatment eligibility versus treatment monitoring; however, given the current role of CD4 [5,18,19], these results remain relevant for both populations. Second, the device does not record data related to the cartridge testing lot or batch number, which could have highlighted batches more prone to invalid results. Third, the type of blood sample used–capillary or venipuncture whole blood–was not known for each test, yet the two sample types have been found to result in different invalid result rates in previous studies [20]. Additionally, due to non-standardization in how operators enter their names, it is possible that some may have been misclassified by testing volume; it was also not possible to determine operator cadre to assess invalid result rates by level of health worker and training as this information was not possible to gather. Furthermore, the definition of health facilities may have differed by country and facilities in the same category may vary in the type of services offered between countries. No study of invalid result rates using conventional CD4 testing platforms has been conducted, limiting our ability to compare invalid result rates between POC and laboratory-based programs. This analysis and comparison is needed for laboratory-based technologies and may suggest that invalid result rates between the two types of technologies may be more similar than often suggested. Finally, the data and results from proficiency testing panels could not be linked to this analysis primarily due to non-electronic data capture of external quality assessments. However, this comparison would be worthwhile and should be considered in the future to strengthen monitoring of POC testing programs.

Despite concerns that POC CD4 testing compromises testing quality [21], this study shows that the scale up of POC CD4 testing using the Alere Pima Analyzer is feasible at a large scale and can be conducted through national POC CD4 testing programs. Previous investments in CD4 infrastructure and testing networks, both laboratory-based and POC, can support ongoing access to identify patients with advanced HIV disease. National testing programs should determine acceptable invalid rates while also considering and weighing the benefit of same-day test result turnaround and faster clinical decisions [10]. Lessons learned from POC CD4 scale-up and invalid test patterns will be useful to inform the scale up of other POC devices, such as early infant diagnosis, viral load testing, hepatitis, and tuberculosis, in the future.
